# Cognitive–Evaluative Dimension of Pain in Neuropathic Pain Relapse in Sciatica: A Case Report

**DOI:** 10.3390/medicina57070658

**Published:** 2021-06-27

**Authors:** Tsubasa Kawasaki, Takuya Yada, Masahiro Ohira

**Affiliations:** 1 Department of Physical Therapy, School of Health Sciences, Tokyo International University, Saitama 350-1197, Japan; 2 Department of Rehabilitation, National Hospital Organization, Tokyo Medical Center, Tokyo 152-8902, Japan; yada51111102@yahoo.co.jp; 3 Department of Rehabilitation, Faculty of Health Science, Uekusa Gakuen University, Chiba 264-0007, Japan; m-oohira@uekusa.ac.jp

**Keywords:** neuropathic pain, pain relapse, sciatica, case report

## Abstract

The cognitive–evaluative (C–E) dimension of pain is commonly observed in patients with a relatively long duration of pain. However, little is known about the effects of pain relapse on the C–E dimension of pain. Moreover, the improvement process of the C–E dimension of pain following treatment is unknown. The objective of this case report was to (a) demonstrate that the C–E dimension was affected in the acute phase of neuropathic pain in cases of pain relapse, and (b) demonstrate the improvement process of the C–E dimension of pain. A woman was diagnosed with low back pain (LBP) and sciatica. The patient had previously experienced symptoms of LBP and sciatica; thus, this episode was a case of pain relapse. At the beginning of rehabilitation, the C–E dimension of pain was present in addition to the sensory–discriminative (S–D) dimension of pain. It was observed that improvement of the C–E dimension of pain was delayed in comparison with that of the S–D dimension of pain. The C–E dimension of pain was observed with pain relapse even though it was in the acute phase of pain. This case provides a novel insight into the C–E dimension of pain. Moreover, the delay in improving the C–E dimension of pain indicates a difference in the improvement process for each pain dimension.

## 1. Introduction

Neuropathic pain can cause both individual and social problems. The severity of neuropathic pain is higher than that of other types of pain and induces a remarkable decrease in the quality of life (QOL) [1,2]. Neuropathic pain is a predominant cause of chronic pain, with a prevalence of 6.5 to 17.9% in the general population [3,4,5,6,7,8]. In particular, young working adults have a greater incidence of neuropathic pain [4,6,7,8], resulting in lost wages and reduced employee productivity. Regarding social problems, O’Connor et al. reported that neuropathic pain is more difficult to treat compared to other types of chronic pain and is therefore associated with higher medical costs [9]. Thus, neuropathic pain can be considered not only an individual’s problem but also a serious social problem [10].

According to the neuromatrix theory of pain [11], prolonged neuropathic pain is especially associated with the affective–emotional (A–E) (e.g., depression, anxiety, and frustration) [12,13,14] and cognitive–evaluative (C–E) (e.g., pain catastrophizing beliefs, altered body image, and neglect-like symptoms) [15] dimensions of pain rather than with the sensory–discriminative (S–D) dimension of pain (e.g., pain intensity, type of pain, and pain location) [16]. Pain catastrophizing belief, which is one of the C–E dimensions of pain, is a critical component of fear-avoidance beliefs. Fear-avoidance beliefs lead to a direct decrease in activities of daily living [17,18,19] and QOL [1]. Thus, enhanced pain catastrophizing beliefs are associated with intractable neuropathic pain. Three different elements of pain catastrophizing beliefs, namely rumination (“I can’t stop thinking about how much it hurts”), magnification (“I worry that something serious may happen”), and helplessness (“There is nothing I can do to reduce the intensity of the pain”) independently affect fear-avoidance beliefs. Thus, pain catastrophizing beliefs are more common in patients with prolonged pain [2].

Another expression of the C–E dimension of pain is the change in body image. Patients often have a malformed body image due to this dimension of pain. For example, in one study, it was observed that patients with complex regional pain syndrome (CRPS) drew a large-sized image of their affected body part [20]. Moreover, Peltz et al. demonstrated that patients with CRPS perceived their affected hand size to be larger than the reality, and the larger estimation was significantly related to the duration of the pain [21]. Conversely, pain intensity in the hands increased when experimental participants observed their enlarged hands through a magnifying mirror [22]. Considering these previous studies, the causal relationship between neuropathic pain or pain intensity and perceived body image needs to be studied, with a specific focus on the assessment of body image, which could indicate the cognitive dimension of pain in patients.

As mentioned earlier, pain catastrophizing beliefs are observed in patients who have experienced neuropathic pain for a relatively long time. However, herein, we present the case of a patient who demonstrated the C–E dimension of pain in the acute phase of low back pain (LBP) and sciatica, using the pain catastrophizing scale (PCS) and line drawing of the patient’s body parts. It should be noted that the pain had relapsed twice; thus, the present case indicated the appearance of the C–E dimension of pain in a patient with pain relapse. Moreover, we assessed changes in the C–E dimension of pain after treatment. The purpose of the present case report is to provide a new understanding of (a) the C–E dimension of pain, and (b) the recovery process of the C–E dimension.

## 2. Case

### 2.1. General Information

The case presented is that of a 45-year-old woman (height, 155 cm; weight, 56 kg; body mass index, BMI 23.0). The patient was a clerical worker who complained of working in a sitting position for a long time (6 h) a day, four times a week. The patient was informed that data from the case would be submitted for publication, and she gave her consent for publication of this report.

### 2.2. Medical History

**Figure 1:**
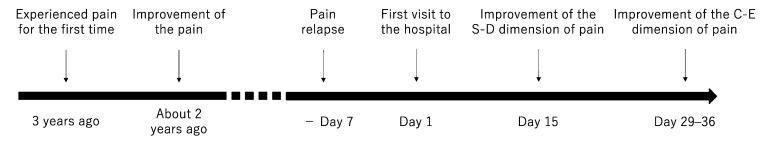
Progress of time points.

The patient was diagnosed with low back pain (LBP) and sciatica (lumbar disc herniation was not observed on magnetic resonance imaging). The patient experienced moderate dull pain from the right lower back to the hip and severe radiating pain in the right lower extremity of the lateral side for a week before her visit to the clinic (- day 7). In addition to the two types of pain, the patient also experienced numbness in the lower extremities. The chief complaint was radiating pain and insufficient sleep due to numbness; thus, the patient hoped that the pain would be relieved and she could sleep well. Another characteristic history was occasionally missing steps on the stairs and on uneven ground. Pregabalin was started at a dose of 150 mg/day. Except for the pain, there was no other medical history. However, it was noteworthy that the patient had experienced pain and numbness three years earlier. At the time, she did not visit any hospital because her symptoms did not worsen as she performed some self-stretching in the bath and could manage the symptoms herself. The symptoms were relieved after approximately a year. A summary of the patient’s pain course is shown in Figure 1.

### 2.3. Assessments Performed Before Rehabilitation on Day 1

**Figure 2:**
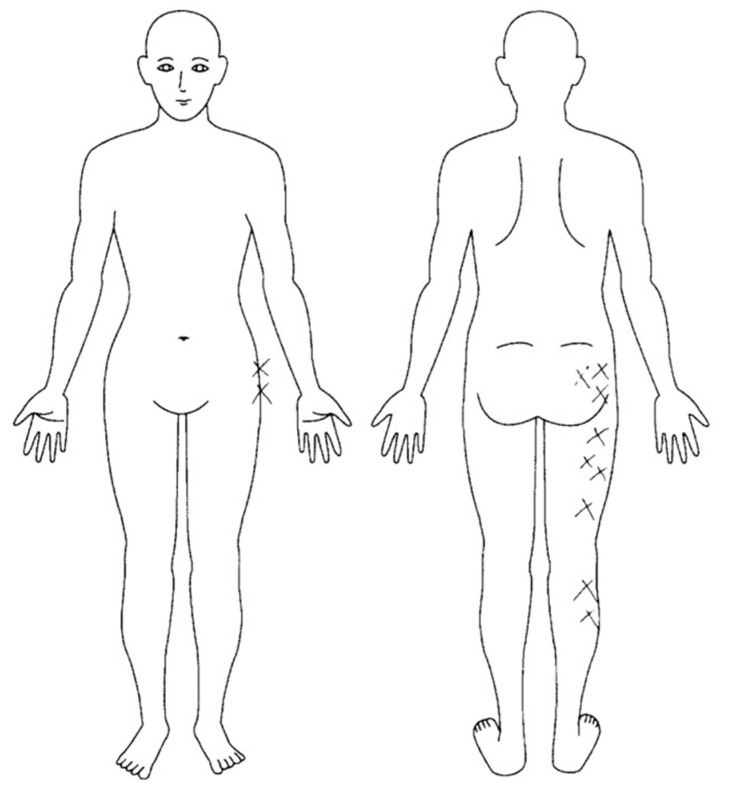
Region of numbness.

Sciatica symptoms (increased numbness and radiating pain in the lateral lower extremity) were prominent in orthopedic tests (Figure 2 and Table 1). Positive signs were observed when a straight leg raising (SLR) test was performed at 30° and on the Bragard test; radiating pain and numbness increased significantly with these tests. In addition, increased radiating pain was registered when Freiberg’s test [23] and the piriformis test [24] were performed; thus, these tests were deemed as positive. Extreme stiffness of the gluteus medius was observed, with muscle tenderness and increased radiating pain. However, muscle weakness was not observed in manual muscle testing.

For the pain assessment, the S–D dimension of pain was assessed using the numerical rating scale (NRS) [25] and the sub-class score in the McGill Pain Questionnaire (MPQ). The scores of the other subclasses in the MPQ were used to assess the A–E and C–E dimensions of pain. The NRS scores for dull and radiating pain were 5 points and 9 points, respectively. Further, the NRS score for numbness was 10 points in the sitting position. The total scores (average score) of the S–D, A–E, and C–E dimensions of pain were 22 (2.2), 2 (0.4), and 3, respectively.

The Japanese version of the PCS [26], which is a translation of the original scale [27], was used to assess the patient’s catastrophizing beliefs involving pain. The patient’s total PCS score was 36 points, which exceeded the cut-off point (30 points) [28]. The total scores (average score) of the sub-class’s rumination, helplessness, and magnification were 18 (3.6), 9 (1.8), and 8 (2.7) points, respectively.

**Figure 3:**
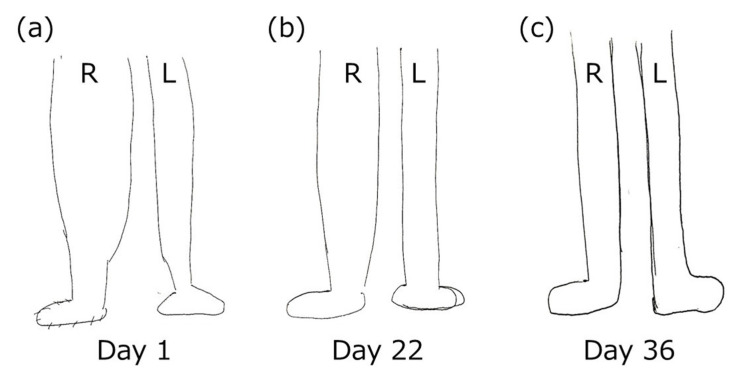
Line drawings of lower extremities in day 1 (**a**), day 22 (**b**), and day 36 (**c**).

A line drawing of the patient’s lower extremity, drawn by herself, is shown in Figure 3. The patient was instructed to draw her legs without looking at them and to not compare the affected side with the unaffected side. The maximum widths and areas of the lower extremity drawings were measured using a Foxit Reader (Ver. 9.3.0, Foxit Japan, Inc. Fremont, CA, USA). The maximum width was 3.84 cm for the right side and 1.81 cm for the left side; i.e., the right side was 2.12 times wider than the left side. Further, the areas were 39.95 cm^2^ for the right side and 21.06 cm^2^ for the left side. That is, the area of the right side was 1.89 times larger than that of the left side (Table 1).

### 2.4. Clinical Diagnosis and Rehabilitation Programs

Radiating pain and numbness were attributed to sciatica, as many of the assessment results (tightness of the piriformis muscle, limitation of the range of motion of external rotation, and positive piriformis and Freiberg tests) indicated sciatica caused by piriformis syndrome. In addition, the reason for the dull pain from the right lower back to the hip was considered to be spasm resulting from sciatica. Therefore, sciatica management was prioritized. To address this issue, physical therapy programs were provided based on the clinical evidence of Last et al. [29], which included stretching and massage of the hamstring and piriformis muscles [30] and manipulation of the lumbar spine [31]. These interventions were provided once per week.

In addition to these programs, the patient received education based on the clinical practice guidelines for chronic pain [32] in the pre-session on day 1. First, explanations about clinical reasoning, intervention plans, and expected changes in pain after treatment were provided. Second, we advised the patient to perform self-exercise training (piriformis muscle stretching, cat and cow stretch, and bending the trunk sideways in the crawling position) daily, as much as possible. Subsequently, the patient performed these training exercises five or six times per week. Furthermore, the patient was instructed to increase physical activity as much as possible to prevent pain catastrophizing and fear-avoidance beliefs by disuse and enhancement of the descending pain inhibitory system [33].

### 2.5. Summary of Change in the Result of Pain-Related Assessments

The results of all assessments are shown in Table 2. A remarkable point about the pain was that there was an incongruency between each dimension of pain. By day 15, symptoms of sciatica disappeared according to the orthopedic tests (i.e., SLR test and Bragard test), and the severity of the S–D dimension in the MPQ, and the degree of dull pain, radiating pain, and numbness, as expressed by the NRS score, were also reduced. However, there was no reduction in the C–E dimension of pain in the MPQ by day 15. After day 15, the pain catastrophizing beliefs slightly improved; however, it should be noted that the PCS score still exceeded the cut-off point of 30. No improvement in the C–E dimension was observed on the patient’s line drawing of her lower extremities. The line drawing of the affected lower extremity did not improve much in comparison with the unaffected side by day 15 but had become similar to that of the unaffected side on day 36. Likewise, there was an improvement in the symptoms of missing the steps on stairs or on uneven ground on day 36; the patient could step optimally.

## 3. Discussion

In this report, we present the case of a patient who demonstrated the C–E dimension of pain in the acute phase. The patient experienced severe pain and numbness due to relapsed sciatica. In addition to the S–D dimension of pain, the C–E dimension of pain was confirmed by the MPQ, PCS, and line drawing portrait during the first session of rehabilitation. However, generally, the C–E dimension of pain occurs when patients experience pain for an unexpectedly long period of time [34,35]. Regarding severe pain and numbness, positive signs were observed on several orthopedic tests, and the radiating pain and numbness in the lateral lower extremity were attributed to sciatica caused by tightness of the piriformis muscle. Based on this clinical reasoning and evidence, we decided upon approaches such as evidence-based physiotherapy and guideline-based medical communication (explanation of clinical reasoning, intervention plan, expected changes, and self-exercise training). The S–D dimension of pain was quickly and notably improved with the rehabilitation intervention, and, as a result, the symptoms of sciatica were almost entirely relieved. Ultimately, the C–E dimension of pain was alleviated after approximately a month of improving the S–D dimension of pain. The appearance of pain and the changes in pain after treatment for each dimension of pain demonstrated some interesting characteristics: (a) no sooner did the patient experience pain than she developed C–E effects of pain, such as pain catastrophizing beliefs and malformed body image, and (b) timing of improvement of the C–E dimension of pain was inconsistent with that of the S–D dimension of pain.

The reason for the C–E dimension of pain during the first session of the rehabilitation was probably because the patient had experienced severe symptoms (radiating pain and numbness) earlier, and the experience influenced the current pain. Specifically, she evaluated the current pain in combination with the memory of her previous pain (pain memory); thus, the pain memory was likely to induce a decreasing pain threshold, such as hyperalgesia [36]. The presence of pain memory has been demonstrated in previous studies in patients with various diseases. For example, Kats and McGuill argued that phantom limb pain in amputees was the result of pain memory prior to amputation [37], and this evidence has recently been reported [38]. Pain memory has also been demonstrated in some patients with implantable cardioverter–defibrillator shocks [39], sickle cell disease [40], and posttraumatic stress disorder following surgery under anesthesia [41]. Many reports have revealed that pain memory affects the appearance and intensity of current pain in patients with multiple diseases. However, little is known about pain memory in patients with LBP and sciatica. Based on this case, we suggest that relapsed LBP is likely associated with pain memory.

The difference in the timing of improvement between the S–D and C–E dimensions of pain indicates that pain is unsynchronized for each dimension of pain. The S–D dimension of pain improved during the first rehabilitation session. However, these improvements were not observed for the C–E dimension of pain (improvements of the C–E dimension of pain were observed about a month later), indicating that improvement of the C–E dimension of pain is delayed in comparison with the S–D dimension of pain. There are two potential explanations for this delay. First, the C–E dimension of pain is difficult to abirritate with only short-term control of the S–D dimension of pain, such as control of pain intensity. Second, considering the changes in pain with treatment, abirritation abolition of the C–E dimension of pain was achieved when the S–D dimension of pain fully improved; for example, the NRS scores recovered to a mild pain level from a severe level. However, these phenomena are still unclear; therefore, additional examination is needed in future research. Importantly, however, the difference in the timing of improvement for the two dimensions of pain was demonstrated in this patient.

The missing of steps on the stairs and on uneven grounds observed in this patient may be associated with the C–E dimension of pain. Luomajoki et al. reported that a decreased ability to discriminate perception (two-point discrimination) and varied estimation of one’s body image results in difficulties in motor control of the affected body part [42]. Additionally, Osumi et al. showed that the experience of pain disturbs the optimal motor control of the hand-reaching movement [43]. In the present patient, the malformed body image and pain-related symptoms might have influenced optimal stepping on the stairs or on uneven ground. The relationship between missing the steps and the C–E dimension of pain is reasonable given that the timing of improvement of this symptom is congruent with that of improvement of the C–E dimension of pain.

There are several limitations to this report that should be investigated in future research. First, this report is a case report, not a study. Therefore, in order to confirm the robustness of these findings, additional investigation with more cases is needed; in particular, according to previous studies, an investigation of gender differences in pain modulation is essential [44,45]. Second, we evaluated the body image using only line drawings; thus, there was a lack of objectivity. Methods such as two-point discrimination must be used for more detachment [46]. Finally, assessments of previous pain episodes were lacking. The current report suggests that the C–E dimension of pain is associated with pain memory due to pain relapse. However, information regarding previous pain was acquired only through medical interviews. Using a cohort design, it is important to verify the causal relationship between the C–E dimension of pain and pain relapse.

## 4. Conclusions

This case indicates the possibility that pain memory could induce the C–E dimension of pain, particularly with relapsed pain in orthopedic disease. This report provides a new understanding of the C–E dimension of pain. Furthermore, the alleviation process of pain implies the necessity of assessing each dimension of pain in a clinical setting.

## Figures and Tables

**Table 1:** Maximum widths and areas in the portrait drawn.

	Day 1		Day 22		Day 36	
	Right	Left	Right	Left	Right	Left
Maximum width in the drawings of the lower extremity (cm)	3.84	1.81	2.95	1.77	1.77	1.55
Ratio of the maximum width (Right/Left) (times)	2.12		1.67		1.14	
Area of the drawings of the lower extremity (cm^2^)	39.95	21.06	34.50	22.12	25.37	23.87
Ratio of the area (Right/Left) (times)	1.89		1.54		1.06	

**Table 2:** Results of physical therapy and pain-related assessments.

	Day 1		Day 8	Day 15	Day 22	Day 29	Day 36
	Pre	Post					
Physical therapy assessments							
ROM							
Hip external rotation (°)	35	45	45	45	45	45	45
Hip flexion (°)	120	120	120	120	120	120	120
SLR (°)	30	55	55	55	60	60	70
Muscle condition							
Stiffness of gluteus medius	(+)	(+)	(+)	(+)	(−)	(−)	(−)
Tenderness of gluteus medius	(+)	(+)	(+)	(+)	(−)	(−)	(−)
Orthopedic tests							
SLR test (positive sign +/−)	(+)	(+)	(+)	(−)	(−)	(−)	(−)
Bragard test (positive sign +/−)	(+)	(+)	(+)	(−)	(−)	(−)	(−)
Freiberg’s test (positive sign +/−)	(+)	(+)	(+)	(+)	(−)	(−)	(−)
Piriformis test (positive sign +/−)	(+)	(+)	(+)	(+)	(−)	(−)	(−)
Pain-related assessments							
MPQ							
Sensory–discriminative dimension (points) *	2.2	0.8	0.8	0.7	0.2	0	0.1
Affective–motivational dimension (points) *	0.4	0.4	0.4	0.4	0.2	0.2	0
Cognitive–evaluative dimension (points) *	3.0	3.0	3.0	3.0	3.0	3.0	2.0
NRS							
Radiating pain	9.0	2.0	3.0	3.0	1.0	1.0	1.0
Numbness	10.0	4.0	5.0	2.0	2.0	2.0	1.0
PCS							
Total (points)	35.0	35.0	34.0	31.0	30.0	25.0	15.0
Rumination (points) *	3.6	3.6	3.4	3.0	2.6	1.8	1.0
Helplessness (points) *	1.8	1.8	1.8	1.6	1.8	1.6	1.0
Magnification (points) *	2.7	2.7	2.7	2.7	2.7	2.7	1.7

Abbreviations: ROM: range of motion; SLR: straight leg raising; MPQ: McGill Pain Questionnaire; NRS: numerical rating scale; PCS: pain catastrophizing scale; * average score.

## Data Availability

Data sharing not applicable. No new data were created or analyzed in this study. Data sharing is not applicable to this article.
